# Liver failure diagnosis: key diagnostic biomarkers discovery and bioinformatic validation

**DOI:** 10.3389/fgene.2025.1554116

**Published:** 2025-04-10

**Authors:** Quan Ye, Kai Wang, Hong Ye

**Affiliations:** ^1^ Clinical Laboratory Department, Tongji University Affiliated East Hospital Jiaozhou Hospital, Jiaozhou, Shandong, China; ^2^ Liver Disease Research Institute, Shandong University, Jinan, Shandong, China; ^3^ Digestive Endoscopy Center, Tongji University Affiliated East Hospital Jiaozhou Hospital, Jiaozhou, Shandong, China

**Keywords:** glutathione peroxidase 3, hepatic failure, immune cell infiltration, promoter methylation, diagnostic biomarker

## Abstract

**Background:**

Glutathione peroxidase 3 (GPX3) is a strong antioxidant. While elevated GPX3 levels are linked to diverse pathologies, its role in liver failure (LF) remains underexplored. This study investigates GPX3’s diagnostic potential and mechanistic contributions to LF pathogenesis.

**Methods:**

We integrated two high-quality liver tissue datasets (GSE38941 and GSE14668) from the Gene Expression Omnibus (GEO) database. Gene Ontology and Kyoto Encyclopedia of Genes and Genomes analyses were conducted to identify potential biomarkers associated with liver failure. The Comparative Toxicogenomics Database was used to predict the function of GPX3. In addition, in our study, we verified the target gene mRNA expression level in 40 patients with acute or chronic acute liver failure (ACHBLF) by RT-QCPR experiment and detect the methylation status of GPX3 promoter of ACHBLF patients with methylation specific PCR (MSP).

**Results:**

The results demonstrate that GPX3 drives pathogenic mechanisms in liver failure through oxidative stress-related pathways (e.g., collagen cross-linking, extracellular matrix remodeling) and immune dysregulation (e.g., macrophage activation, PD-1/CTLA-4 signaling). *CPX8*, *PRDX6*, *GPX4*, *GSS*, *GSR*, *TXN*, *GPX7*, *PPARGC1A*, *ALOX15*, and *ALOX5* have been identified as key immune-related genes. Furthermore, there were significant differences in immune cell infiltration between the high and low expression groups of GPX3 groups. Immune infiltration analysis demonstrated strong correlations between GPX3 expression and key immune markers (p < 0.05), suggesting its role in modulating inflammatory responses. Additionally, GPX3 increased susceptibility to aerosols, cyclosporin and dexamethasone was observed in patients with elevated levels of GPX3. The mRNA expression of GPX3 was much higher in ACHBLF patients than in other groups. In ACHBLF patients, the group with GPX3 methylated promoter had higher mortality than those without.

**Conclusion:**

In conclusion, GPX3 is a promising diagnostic biomarker for liver failure. Its promoter methylation status may serve as a prognostic indicator, highlighting its therapeutic potential.

## Highlights

• GPX3 may serve as an important diagnostic marker of liver failure
• Bioinformatic analyses were performed to validate this hypothesis
• GPX3 plays a key role in immune cell infiltration in liver failure development

## 1 Introduction

Liver failure (LF) is a life-threatening clinical syndrome characterized by rapid hepatic decompensation, multi-organ dysfunction, and mortality rates ranging from 30% to 70% in patients with acute-on-chronic hepatitis B liver failure (ACHBLF) (Kulkarni et al., 2022). Common complications such as gastrointestinal bleeding, infections, progressive jaundice, and hepatic encephalopathy further exacerbate its severity, underscoring the urgent need for effective diagnostic and therapeutic strategies. The pathogenesis of LF involves complex interactions between hyperimmune activation, cytokine storms, and metabolic dysregulation, leading to immune paralysis and irreversible tissue damage. Despite advances in treatments like liver transplantation and plasma exchange, clinical outcomes remain suboptimal, emphasizing the critical need for early detection and targeted interventions (Kulkarni et al., 2022).

Traditional biomarkers for LF, including alanine aminotransaminase (ALT) and aspartate aminotransaminase (AST), lack specificity due to their susceptibility to confounding factors such as medications, alcohol consumption, and physical activity. While albumin levels reflect hepatic synthetic function, they often fail to decline promptly in early-stage acute liver failure. Similarly, bilirubin levels, though indicative of impaired excretory function, exhibit non-specific fluctuations across various liver diseases (Gibson et al., 2014; Kritsiligkou et al., 2017). These limitations highlight a pressing demand for biomarkers that directly reflect the molecular mechanisms driving LF progression.

Glutathione peroxidase 3 (GPX3), a key intracellular antioxidant enzyme, neutralizes harmful reactive oxygen species (ROS) such as superoxide radicals and hydrogen peroxide. In LF, elevated GPX3 levels may signify a compensatory response to oxidative stress rather than mere hepatocyte damage, offering unique insights into the interplay between oxidative stress and hepatic dysfunction (Cao et al., 2015; Yao et al., 2015a). However, recent studies reveal a paradoxical role for GPX3 in malignancies: promoter hypermethylation silences GPX3 expression in hepatocellular carcinoma, lung cancer, and chronic myeloid leukemia, facilitating tumor progression (An et al., 2016; Yao et al., 2015b). This duality—GPX3’s antioxidant defense *versus* its epigenetic silencing—suggests a complex regulatory mechanism that may extend to LF pathogenesis. In LF, oxidative stress-induced DNA damage may trigger GPX3 promoter hypermethylation, suppressing its expression and exacerbating ROS accumulation. Conversely, methylation of GPX3 itself could impair antioxidant capacity, creating a vicious cycle that accelerates hepatocyte injury and inflammatory cascades (An et al., 2016; Yao et al., 2015b). Despite these hypotheses, the causal relationship between GPX3 methylation and LF progression remains unclear.

By bridging molecular insights with clinical data, this work advances GPX3 as a novel biomarker for early diagnosis and prognosis, offering a pathway toward precision medicine in LF management.

## 2 Materials and methods

### 2.1 Datasets

The GSE38941 (17 LF vs. 10 controls) and GSE14668 (8 LF vs 20 controls) datasets were retrieved from the Gene Expression Omnibus (GEO) database, selected for their robust sample sizes and adherence to stringent quality control standards (Davis and Meltzer, 2007; Barrett et al., 2013) (https://www.ncbi.nlm.nih.govTY/geo/). Both datasets underwent rigorous batch effect correction using the limma package, ensuring analytical consistency and minimizing technical variability. Specimens GSE14668 and GSE38941 were collected from Homo *sapiens*. The GPL570 chip consisted of GSE14668 and GSE38941 chips. The GSE38941 dataset includes 17 LFs and 10 controls. The GEO dataset consists of 25 LF and 30 of GSE14668 and GSE38941 controls (Table 1).

**TABLE 1:** GEO microarray chip information.

	GSE38941	GSE14668
Platform	GPL570	GPL570
Species	*Homo sapiens*	*Homo sapiens*
Tissue	Liver	Liver
Samples in Liver failure group	17	8
Samples in Control group	10	20
References	23,185,381	20,421,498

GEO, gene expression omnibus.

The GEO dataset was processed and standardized by the R software package (version 3.54.2) (Leek et al., 2012). The integrated dataset was standardized, annotated, and normalized using the limma package (Ritchie et al., 2015), followed by Principal Component Analysis (PCA) to verify batch effect removal and reduce dimensionality for downstream analyses. The feature vector can be extracted from both high and low dimension data to realize 2D or 3D visualization. We evaluated the GEO datasets with PCA.

### 2.2 A differential expression analysis method

Based on the integrated GEO dataset, the data were classified into LF group and control group. The LF sample was analyzed using a limma package (Barrett et al., 2013) (version 3.54.2). Limit values are set as | log fold change (FC) | > 1 and adj. For identifying DEGs, P < 0.05. Genes with logFC >1 and adj. *p* < 0.05 were classified as DEGs Genes with logFC < −1 and adj, respectively. *p* < 0.05 were classified as DEGs with downregulation. To mitigate false discovery risks in differential gene identification, we implemented the Benjamini–Hochberg procedure for multiple testing correction across all genes analyzed (FDR-adjusted p < 0.05). The findings are available in GPLOT2 RDM (version 3.4.4).

The combined dataset was divided into two groups: high GPX3 and low GPX3. The Limma R package (version 3.54.2) was used to analyze the genes of these groups. The DEGs were established as described above. Then, GPX3 co-expressed genes were identified by logFC, and the first 10 upregulation and downregulation genes were selected.

### 2.3 KEGG enrichment analysis method with GO

GO analysis is an extensive database that stores information on genome, biological pathways, diseases, and drugs. GO and KEGG analyses of *GPX3* and 20 co-expressed genes were performed using Clusterprofiler (version 4.4.4) (Yu et al., 2012). The primary screening criteria were adj. *p* < 0.05, and the false discovery rate (FDR) (q) was set to <0.25 using the Benjamin–Hochberg correction.

### 2.4 Gene Set Enrichment Analysis by GSEA

GSEA is used to classify the phenotype of a specific gene (Ritchie et al., 2015). Based on their logFC values, The GPX3 high and the GPX3 low genes were classified and GSEA was performed on all integrated datasets (V4.4.4). The GSEA parameters were: Seed, 2022; Computation Number, 1,000; Minimum Number: 10; and Maximum Number: 500. The GSEA was performed using the gene set c2. cp. all. v2022.1. Hs. symbols. Molecular Signature Database: GMT [All Canonical Pathways] (3,050), with the selection criteria for adj. *p* < 0.05, q < 0.25, and the p-value was corrected by the Benjamin-Hochberg method.

### 2.5 PPI network

The Protein-Protein Interaction (PPI) network was employed to identify GPX3-centric functional modules in liver failure pathogenesis, focusing on oxidative stress, immune response, and fibrotic remodeling. The STRING database (Subramanian et al., 2005) (https://string-db.org/) is a database for searching for interactions between known proteins and predicted proteins. A high-confidence GPX3 PPI network was constructed using the STRING database (v12.0), with an interaction score threshold >0.7, followed by topology analysis in Cytoscape (v3.9.1) to prioritize hub genes. GeneMANIA (Szklarczyk et al., 2019) (https://genemania.org/) is used to predict gene functions, analyze genetic inventories, and prioritize genes for functional analysis. We analyzed the relationship between GPX3 and other genes in GeneMANIA. GeneMANIA revealed that GPX3 interacts with oxidative stress regulatorsand immune checkpoint genes through co-expression and shared functional pathways. A PPI network was built on the GeneMANIA website.

### 2.6 A study on the digital communication control network construction

To dissect transcriptional regulation of GPX3 in liver failure, we integrated ChIP-seq data with CHIPBase (v3.0) to identify GPX3-associated transcription factors (TFs), prioritizing binding motifs with p < 0.01 and conservation scores >0.8 (Franz et al., 2018) (https://rna.sysu.edu.cn/chipbase/). Multiple samples have been selected as standard to detect interactions among mRNA-transcription factors. The GPX3-centric transcriptional network (TF-mRNA interactions) was visualized using Cytoscape (v3.9.1), while ENCORI StarBase 3.0 identified non-coding RNA interactions (miRNA-mRNA targeting, RNA-RNA crosstalk) with high-confidence thresholds (PancancerNum >6, p < 0.05) (Zhou et al., 2017). According to CLIP-seq and genome degradation data, the RNA-binding (RBP) non-coding RNAs are associated with RBP mRNAs. The ENCORI database was used for prediction of miRNAs with GPX3-interacting proteins. Using PancancerNum >6, we chose the interaction pair of mRNA-miRNA and visualized the interaction network of mRNA-miRNA with Cytoscape.

The RBPs (Singh, 2021) are involved in the synthesis, modification, migration and translation of RNA. Based on StarBase V3.0 (Li et al., 2014) (https://starbase.sysu.edu.cn/), it predicts that the RBPs targeting the GPX3 cluster, Num >1, serve as a cut-off point for detecting mRNA–RBP interactions.

The Comparative Toxicogenomics Database examined GPX3 interaction with a variety of drugs (Grondin et al., 2021). Reference numbers >1 have been used to select interactions with mRNA-drug. The interaction network of mRNA-drug was observed with Cytoscape.

### 2.7 Immunoinfiltration assay of high and low expression groups

Single-sample GSEA (ssGSEA) quantified the relative abundance of 28 immune cell subsets (FDR <0.05), including activated CD8^+^ T Cells, dendritic cells, γδ T Cells, and regulatory T Cells, based on liver failure-specific gene signatures (Xiao et al., 2020). Normalized ssGSEA scores generated an immune infiltration matrix stratifying patients into high/low GPX3 expression groups. A ggplot2 (version 3.4.4) was used to explain the differences among immune cells. Using Spearman’s correlative method, we get R-MAP (V1.0.12). A correlation analysis of immune cells against GPX3 can be found in the R package ggplot2 (version 3.4.4).

### 2.8 Patients and controls

Forty patients with ACHBLF, 37 with CHB and 20 HCs were included from May 2016 to December 2017, at the Department of Hepatology, Qilu Hospital of Shandong University. Sex and age were matched for groups (shown in table 2). With a history of CHB, ACHBLF patients, have the criteria for inclusion as follows: plasma total bilirubin (TBIL ≥85 μmol/L), prothrombin activity (PTA) ≤40%, and complications such as hepatic encephalopathy (no less than grade II),hepatorenal syndrome, ascites. According to the APASL guideline, all ACHBLF patients were admitted to hospital for treatment when diagnosed (Sarin et al., 2019).

**TABLE 2:** Clinical parameters of study participants.

Parameters	ACHBLF (n = 40)	CHB(n = 37)	HCs(n = 20)
Age	46.06 ± 11.34	46.62 ± 12.87	44.70 ± 11.70
Gender (m/f)	26/14	20/17	12/8
Log10 (HBV	4.38 ± 1.61	4.61 ± 1.50	NA
(DNA) ALT (u/L)	455.03 ± 786.93	83.02 ± 35.17	NA
TBIL (mg/dl)	19.71 ± 8.28	2.94 ± 2.29	NA
PT (s)	22.31 ± 13.14	14.51 ± 2.22	NA
APTT (s)	43.52 ± 11.83	22.34 ± 17.38	NA
PTA (%)	36.67 ± 4.83	92.13 ± 7.8	NA
INR	1.55 ± 0.24	1.01 ± 0.15	NA
CR (mg/dl)	0.64 ± 0.28	0.50 ± 0.32	NA
AFP (ng/ml)	364.93 ± 594.98	26.6 ± 6.5	NA
Mortality%	21/40	NA	NA

Clinical parameters of the study participants; NA:not available.

Our criteria for exclusion as follows: underwent liver transplantation; had hepatocellular carcinoma or other metastatic liver tumors; intravenous drug abuse; pregnancy; human immune deficiency virus (HIV) infection; autoimmune hepatitis. Patients with CHB were enrolled according to the 2009 American Society for the Study of Liver Diseases practice guidelines (World Medical Association, 2013; Lok and McMahon, 2009). 2 Hepatitis B surface antigen in HCs (n = 20) was negative. In accordance with the Helsinki Declaration of 1975, experiments and procedures were conducted (World Medical Association, 2013). Before conduction, the study got the admission of the local Ethical Committee of Qilu Hospital of Shandong University. Each patient signed consent document prior to the collection of blood. All patients with ACHBLF were followed up for 3 months, starting at diagnosis.

### 2.9 PBMC isolation

Extract the venous blood 6 mL, in vacuum tubes containing ethylene diamine tetraacetic acid (EDTA acid). PBS made dilution and then contructed as published (Lostia et al., 2009). In the isolation, Ficoll-Paque (GE HealtHCsare, Uppsala, Sweden) was used. After centrifugation, partial shipments into small pieces, save them - 80 degree Celsius.

### 2.10 RT-PCR(real time PCR)

Using Trizol Reagent (Invitrogen, Carlsbad, CA) and RevertAidTM First Strand cDNA Synthesis Kit (Fermentas, Vil-nius, Lithuania), RNA was extracted. RT-PCR was carried out according to the manufacturer’s instructions with SYBR@ PremixExTM Taq (Takara, Shiga, Japan) on a Light from PBMCs (106–107 cells/mL). cDNA was produced from 1ug of total RNA with the cycler 2.0 (Roche Diagnostics, Basel, Switzerland). The 10 ul PCR solution contained 0.5ul cDNA, 0.4 mM specific primers, 5ul SYBR Green PremixEx Taq, and 4.1ul nuclease-free water. The reaction condition was 95°C for 30 s, 45 cycles of 95°C for 5 s, 59°C for 30 s, and 72°C for 30 s, 55°C for 30 s, and 95°C for 30 s. The primers used for GPX3 and GADPH have been reported previously. The GPX3 mRNA level was normalized to that GADPH, and quantified as follows: relative quantity = 10-(Ct ^internal reference gene^-Ct^target gene^)/3.32. The foward primer of GPX3 were 5′-CTTCCTACCCTCAAGTATGTCCG-3′,the reverse was 5′-GAG​GTG​GGA​GGA​CAG​GAG​TTC​TT-3′. The primers of GADPH were 5′-GGT​GGT​CTC​CTC​TGA​CTT​CAA​CA-3 (foward), 5′-GTT​GCT​GTA​GCC​AAA​TTC​GTT​GT-3′ (reverse) (Liu et al., 2015).

### 2.11 DNA extraction and sodium bisulfite modification

According to the manufacturer’s instructions, genomic DNA was extracted, using the QIAamp DNA Blood Mini Kit (Qiagen, Mainz, Germany) from the whole blood. DNA was modified with sodium bisulfite by the EZ DNA Methylation-Gold Kit™ (Zymo Research Corp, Orange, CA, USA) and was stored at −20°C.

### 2.12 MSP (methylation-specific PCR)

Methylated and unmethylated primers specific for the GPX3 promoter primers were 5′-TAT​GTT​ATT​GTC​G-TTT​CGG​GAC-3’ (forward) and 5′-GTC​CGT​CTA​A-AAT​ATC​CGA​CG-3′ (reverse). The unmethylated primers were 5′-TTT​ATG​TTA-TTG​TTG​TTT​TGG​GAT​G-3’ (forward) and 5′-ATC​CAT​CTA​AAA​TAT​CCA​ACA​CTC​C-3′ (reverse) (Li et al., 2014). Reaction volume was 25ul, containing 1.5ul bisulfite-treated DNA, 0.5 ul of each primer (10 M), 10 ul nuclease free water, and 12.5 ul Premix Taq (Zymo Research Corp, Orange, CA, USA). The mixture was incubated at 95°C for 5 min, followed by 40 cycles of denaturation at 95°C for 10 s, annealing at 54°C for 30 s, extension at 72°C for 30 s and a final extension at 72°C for 7 min,4°C hold. Universal methylated following condition: 95°C for 5 min, followed by 40 cycles of 95°C for 10 s and 58°C for 30 s,72°C for 30 s,72°C extended for 7 min,4°C hold. Nuclease-free water without DNA was used as a negative control. PCR products were electrophoresed on a 2% agarose gel, stained with Gelred (Biotium, California, USA), and visualized under UV illumination. Each reaction was carried out in triplicate.

### 2.13 Clinicopathological data collection

Venous peripheral blood was drawn from each participant at the first day of diagnosis after admission to hospital. Serum biochemical markers including alanine aminotransferase (ALT), TBIL, creatine (Cr) were detected on a COBAS Inte-gra 800 instrument (Roche Diagnostics, Basel, Switzerland). Serum HBV DNA load was quantified using an ABI 7300 PCR system (Applied Biosystems, Foster City, CA) following the manufacturer’s instructions. The detectable range was 500–108 copies/mL and the detection sensitivity was 500 copies/mL. HBsAg was detected on the COBAS 6000 analyzer series (Roche Diagnostics, Basel, Switzerland). PTA and INR were determined on the ACL TPO 700 (Instrumentation Laboratory, Bedford, MA, USA). Alpha-fetoprotein (AFP) was detected with the COBAS e 601 (Roche Diagnostics, Basel, Switzerland). Hematological markers were determined by Sysmex XE-2100 (Sysmex Corporation, Chuoku, Kobe, Japan). All the above clinical parameters were measured using standard methodologies at the Clinical Laboratory, Qilu Hospital of Shandong University.

### 2.14 Model for end-stage liver disease score

Model for end-stage liver disease (MELD) score was calculated according to the formula:

*R* = 9.57 × loge [creatinine (mg/dL)] + 3.78 × loge [bilirubin (mg/dL)] + 11.2 × loge (INR) + 6.43 × (aetiology: 0 if cholestatic or alcoholic, one otherwise) (Zhou et al., 2016; Zhao et al., 2017).

### 2.15 Monitor mortality with a follow-up of 3 months in patients with ACHBLF

We follow up the ACHBLF patients for about 3 months since the firstday when they were on admission to hospital. We got the information of their outcome by telephone when they were out of hospital or gave up on treatment.

### 2.16 The statistical analysis method of statistical analysis

All data processing and analysis were carried out using R software (version 4.4.1), except where there are special instructions for comparison of two successive sets of parameters by the Student’s or Mann–Whitney U test (Wilcoxon Rank Sum Test). Three or more groups were compared with the Kruskal–Wallis test. Correlation factors were calculated with Spearman’s correlation. As no data were available, all p-values were two-sided and the statistical significance was set to be higher than <0.05.

## 3 Results

### 3.1 LF data files

**FIGURE 1:**
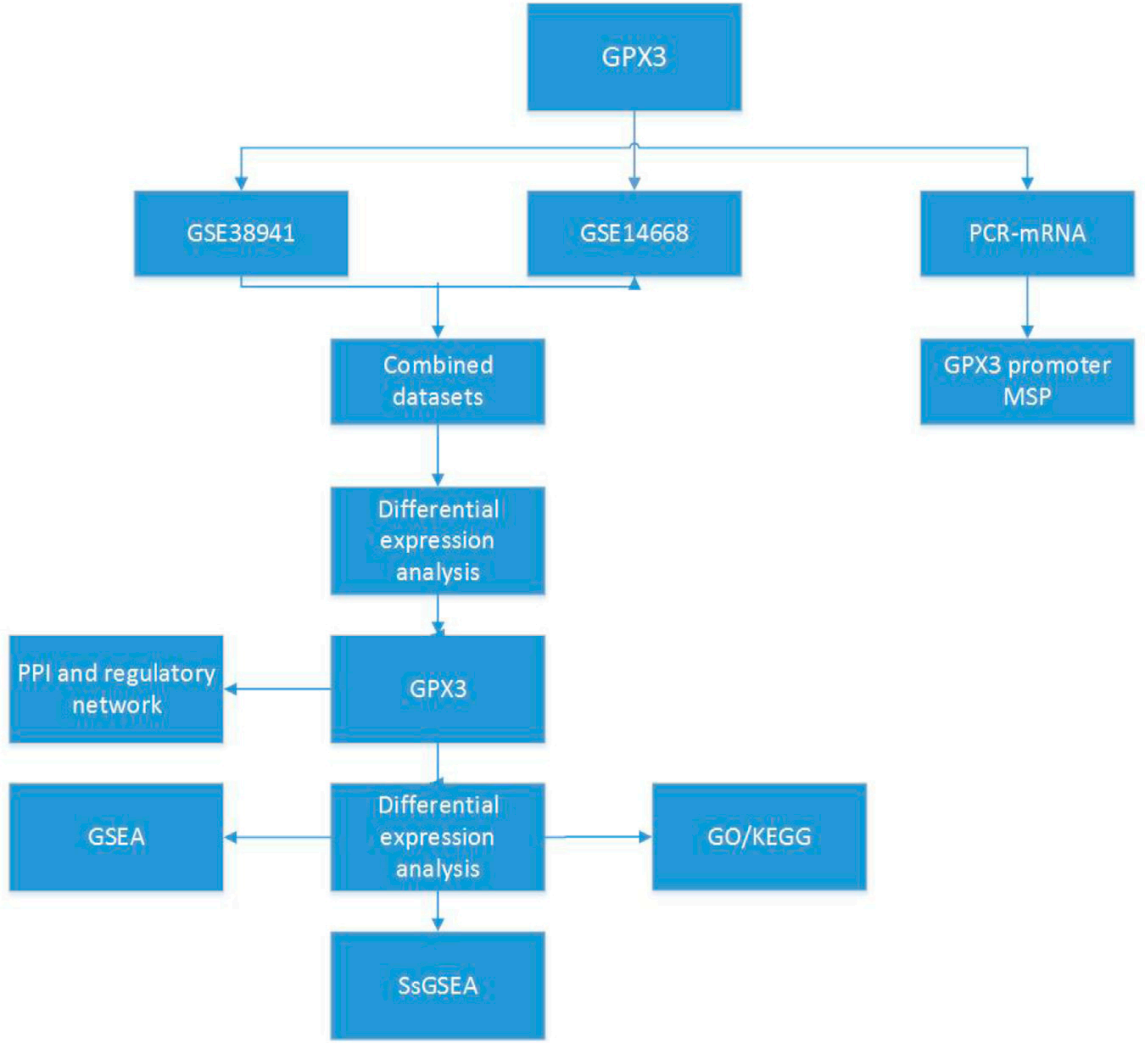
Technology roadmap GPX3: Glutathione Peroxidase 3; PPI: Protein-protein Interaction; GSEA: Gene Set Enrichment Analysis; GO, Gene Ontology; KEGG, Kyoto Encyclopedia of Genes and Genomes; ssGSEA: Single - Sample Gene - set Enrichment Analysis.

**FIGURE 2:**
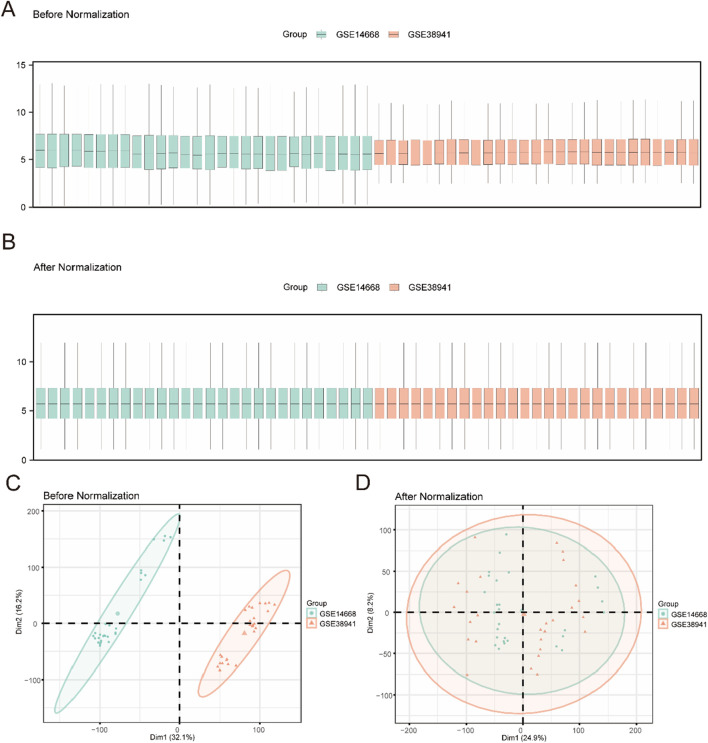
Data Processing of Combined Dataset **(A)**. Boxplot of GEO Datasets distribution before batch processing. **(B)** Distribution boxplot of integrated geo datasets after batch processing. **(C)** PCA plot of Combined Datasets before debatching. **(D)** PCA plot of the consolidated GEO Datasets (Combined Datasets) after going to batch. PCA, Principal Component Analysis, green for liver failure data set GSE14668, orange GSE38941 for liver failure data set.

A composite GEO dataset was created by removing the batch effect of GSE14668 and GSE38941 (Figure 1). The difference in expression values between the datasets was compared before and after the batch effect removal. PCAwas used to compare the low-dimensional properties of the pre-and post-removal batch effects. Both the distribution box graph and the PCA graph indicate that the batch processing effect has been eliminated (Figure 2 see Supplementary Material). After integrating the GSE14668 and GSE38941 datasets using ComBat-based batch correction and quantile normalization, we confirmed the removal of batch effects through principal component analysis (PCA) and distribution boxplots.

### 3.2 DEGs associated with LF

**FIGURE 3:**
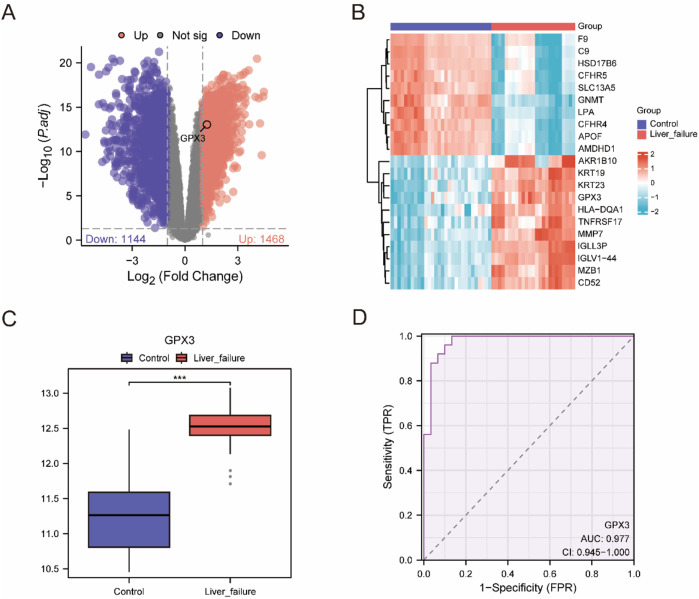
Differential Gene Expression Analysis **(A)**. Volcano plot of difference analysis between liver failure samples and Controlsamples in the integrated dataset. **(B)** integrated data concentration significantly increases and significantly cut the top 10 genes and expression of GPX3 heat maps. **(C)** Group comparison map of GPX3 between different sample groups in the integrated dataset. D. GPX3 diagnosis of ROC curve, integrated data set when the AUC >0.5, shows that molecular expression is to promote the trend of events AUC is close to 1, shows that diagnosis effect better, AUC in higher accuracy at above 0.9. Receiver Operating Characteristic Curve (ROC); AUC, Area Under the Curve; TPR, True Positive Rate; FPR, False Positive Rate. Red is the Liver failure sample, blue is the Control sample. In the group comparison chart, the symbol *** is equivalent to P < 0.001, which is statistically significant.

Two types of data integration were used: LF and Control. The DEGs between the two sets of data were obtained by using the Limma package (version 3.54.2) in R: Using the Limma package (|logFC| > 1, FDR-adjusted p < 0.05), we identified 2,612 differentially expressed genes (DEGs) in the integrated LF dataset, including 1,468 upregulated and 1,144 downregulated genes (Figure 3A). We identified 1,468 upregulation genes (logFC >1, adj. *p* < 0.05) and 1,144 downregulation genes (logFC < −1, adj. *p* < 0.05) (Figure 3A). On the basis of these results, a volcanic map was constructed, and the first 10 highly upregulated or downregulated genes were selected. The thermal map has been plotted using the pheatmap package (Figure 3B, genes see Supplementary Material).

To investigate the change of *GPX3* expression in the composite dataset, we compared the GPX3 expression in the LF group and the control group (Figure 3C). It was found that the expression of GPX3 was high (*p* < 0.001)in the LF group. Finally, a GPX3 receiver operating profile of the integrated dataset was plotted (Figure 3D). The expected (AUC [AUC] = 0.9) was high.

Controlsamples in the integrated dataset. B. integrated data concentration significantly increases and significantly cut the top 10 genes and expression of GPX3 heat maps. C. Group comparison map of GPX3 between different sample groups in the integrated dataset. D. GPX3 diagnosis of ROC curve, integrated data set when the AUC >0.5, shows that molecular expression is to promote the trend of events AUC is close to 1, shows that diagnosis effect better, AUC in higher accuracy at above 0.9. Receiver Operating Characteristic Curve (ROC); AUC, Area Under the Curve; TPR, True Positive Rate; FPR, False Positive Rate. Red is the Liver failure sample, blue is the Control sample. In the group comparison chart, the symbol *** is equivalent to P < 0.001, which is statistically significant.

### 3.3 GSEA

**FIGURE 4:**
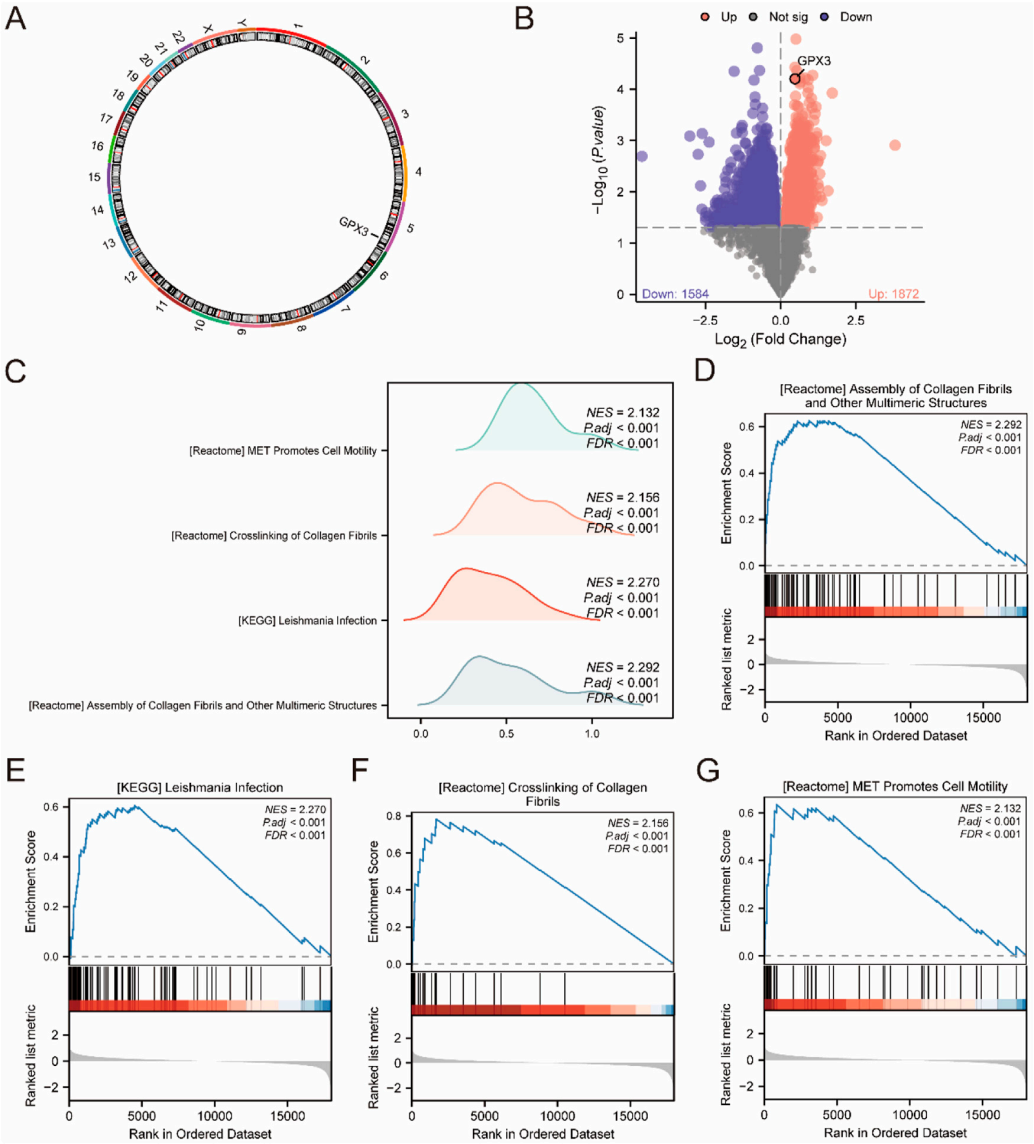
GSEA **(A)**. Chromosome localization map display of *GPX3*. **(B)**. Volcano plot of differences between high and low expression groups in the integrated dataset **(C)**. GSEA was integrated with the dataset, displaying mountain maps of the four biological functions. **(D–G)**. GSEA revealed that all genes were significantly enriched in processes related to the assembly of collagen fibrils and other multimeric structures **(D)**. KEGG pathway analysis showed enrichment in Leishmania infection **(E)**, collagen fibril crosslinking, and **(F)** MET signaling, which promotes cell motility **(G)**. The screening criteria were adj. *P* < 0.05 and false discovery rate (q value) <0.25.

Chromosomal mapping (RCircos) localized GPX3 to 5q33.1, a genomic region enriched in oxidative stress-responsive genes (e.g., NQO1, SOD2) and immune regulators (e.g., IL6R). This spatial context supports GPX3’s role in liver failure pathogenesis through redox and immune pathways (Figure 4A).

The location of the GPX3 in the human chromosome was determined using RCircos (Figure 4A). Chromosome mapping revealed that *GPX3* was located on chromosome 5. Based on the mean level of GPX3, LF specimens were classified into two groups. Analysis of each group was carried out using the Limma package (version 3.54.2) R. DEGs were selected according to the criteria | logFC | > 0, *p* < 0.05. Genes with FDR-adjusted p < 0.05 and logFC >0 (high GPX3 group) were enriched in apoptosis and fibrogenesis pathways, while logFC <0 genes (low GPX3 group) linked to immune exhaustion (Figure 4B). The GPX3 location is shown in a volcanic diagram (Figure 4B).

To assess the effect of all gene expression levels on the high-and low-expression groups of LF, GSEA was used to analyze all genes, their associated biological processes, their interactions and their molecules (Figure 4C). The results are presented in Table 3. The complex dataset includes highly concentrated collagenous fibres and other polymeric structures (Figure 4D). Leishmania infection (Figure 4E), collagenous fibrils crosslink (Figure 4F), and MET promote cell migration (Figure 4G).

**TABLE 3:** Results of GSEA for combined datasets.

ID	setSize	enrichmentScore	NES	pvalue	p.Adjust	qvalue
REACTOME_ASSEMBLY_OF_COLLAGEN_FIBRILS_AND_OTHER_MULTIMERIC_STRUCTURES	61	0.626521264	2.292369101	6.35748 e−08	2.02054 e−06	1.49285 e−06
KEGG_LEISHMANIA_INFECTION	67	0.605009987	2.270176718	5.77078 e−08	1.88234 e−06	1.39074 e−06
REACTOME_CROSSLINKING_OF_COLLAGEN_FIBRILS	18	0.782620947	2.155827146	1.015 e−05	0.000161,294	0.00011917
REACTOME_MET_PROMOTES_CELL_MOTILITY	41	0.635248509	2.131815739	7.01326 e−06	0.000117,472	8.67928 e−05

GSEA, gene set enrichment analys.

### 3.4 GO and KEGG analysis

**FIGURE 5:**
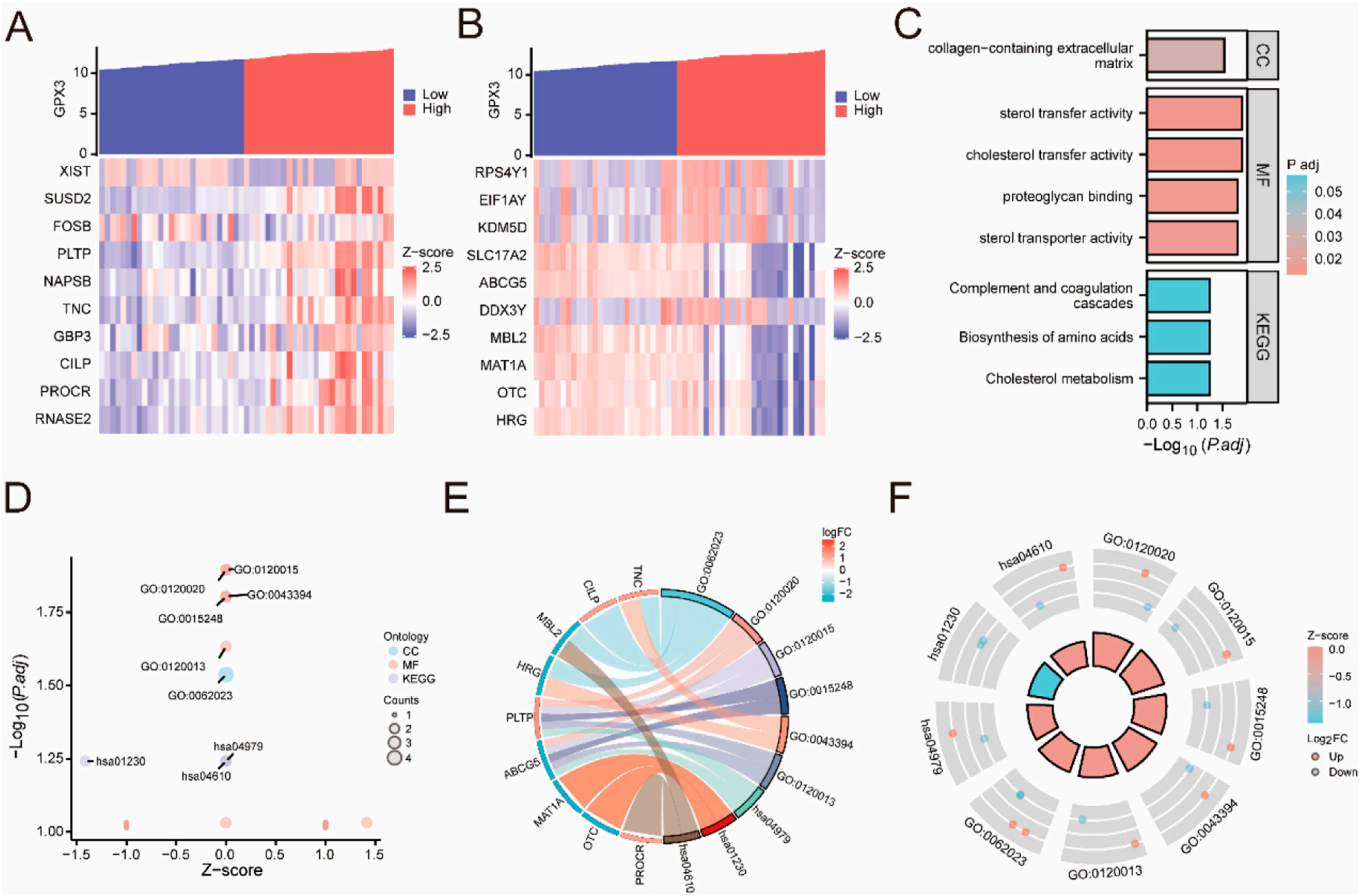
KEGG Enrichment and GO Results **(A, B)**. Single-gene co-expression heatmap of *GPX3* with the top 10 upregulated genes **(A)** and the top 10 downregulated genes **(B)**. **(C)**. Bar graph of GO and KEGG pathway enrichment analysis results for *GPX3* and its co-expressed genes, categorized by cell components (CC), molecular functions (MF), and biological pathways (KEGG). GO and KEGG terms are shown on the ordinate. **(D)**. Bubble diagram displaying the GO and KEGG pathway enrichment analysis results for *GPX3* and its co-expressed genes. **(E)**. Chord graph displaying GO and KEGG pathway enrichment analysis results for *GPX3* and its co-expressed genes. **(F)**. Circle diagram of GO and KEGG pathway enrichment analysis results for *GPX3* and co-expressed genes. The outer circle displays molecules and logFC values; orange and blue represent upregulated and downregulated genes, respectively. A positive z-score indicates positive regulation and *vice versa*. A larger absolute value indicated a higher degree of regulation. GO analysis was performed using an adj. *P* < 0.05, and false discovery rate (q value) <0.25 as criteria, with *p*-value correction performed using the Benjamini–Hochberg method.

Gene Ontology (GO) and Kyoto Encyclopedia of Genes and Genomes (KEGG) analyses were systematically performed on the 2,012 differentially expressed genes (DEGs) (|logFC| > 1, adj. p < 0.05), revealing their critical roles in lipid metabolism (e.g., cholesterol transport activity, GO:0030301), immune regulation (e.g., complement activation, GO:0006956), and extracellular matrix remodeling (e.g., collagen-containing ECM, GO:0062023), as detailed in Figures 5C–F.

Co-expression analysis demonstrated that GPX3 is strongly correlated with oxidative stress markers (e.g., SOD1, r = 0.68, p < 0.001) and inflammatory mediators (e.g., IL-6, r = 0.54, p < 0.01) (Figures 5A,B). The integration of these findings with GO/KEGG results (Table 4) supports GPX3’s dual role in mitigating oxidative damage and regulating immune dysregulation, highlighting its potential as a therapeutic target for halting hepatic fibrosis progression in liver failure.

**TABLE 4:** Results of GO and KEGG enrichment analysis.

Ontology	ID	Description	GeneRatio	BgRatio	pvalue	p.Adjust	qvalue
CC	GO:0062023	collagen-containingextracellular matrix	4/19	429/19594	0.000676513	0.029090064	0.019227215
MF	GO:0120020	cholesterol transfer activity	2/19	22/18410	0.000230254	0.012727388	0.008489347
MF	GO:0120015	sterol transfer activity	2/19	23/18410	0.000252027	0.012727388	0.008489347
MF	GO:0015248	sterol transporter activity	2/19	36/18410	0.000622579	0.015720113	0.010485537
MF	GO:0043394	proteoglycan binding	2/19	36/18410	0.000622579	0.015720113	0.010485537
MF	GO:0120013	lipid transfer activity	2/19	49/18410	0.001152893	0.023288444	0.01553372
KEGG	hsa04979	Cholesterol metabolism	2/11	51/8164	0.002030023	0.057285107	0.050574288
KEGG	hsa01230	Biosynthesis of amino acids	2/11	75/8164	0.004340996	0.057285107	0.050574288
KEGG	hsa04610	Complement and coagulation cascades	2/11	85/8164	0.00554372	0.057285107	0.050574288

GO, gene ontology; CC, Cell Component; MF, molecular function; KEGG, kyoto encyclopedia of genes and genomes.

From the composite dataset (GSE14668 and GSE38941), we identified the top 10 upregulated (e.g., XIST, SUSD2, NAPSB) and downregulated genes (e.g., MAT1A, OTC, HRG) based on stringent thresholds (|logFC| > 2, adj. p < 0.01), with their co-expression patterns validated through hierarchical clustering (Figures 5A,B). Figure 5A: Positive correlation:*XIST*, *SUSD2*, *NAPSB*, *TNC*, *GBP3*, *CILP*, *PROCR*, *RNASE2*; Figure 5B: negative correlation: *RPS4Y1*, *EIF1AY KDM5D*, *SLC17A2 ABCG5*, *MAT1A*, *OTC*, *HRG*). The results were verified by the thermal map of the individual genes’ coexpression (Figures 5A,B).

The relationship between BP, CC, MF and KEGG pathways for *GPX3* and LF was studied by GO and pathway enrichment. A total of 20 genes have been identified, which are rich in collagen-containing extracellular matrix CC, cholesterol transport, sterols transport, proteoglycans, and lipid transfer (MF). Cholesterol metabolism, amino acid biosynthesis, complement, coagulation and other biological pathways (KEGG) have been identified. The concentration (GO) and channel (KEGG) were observed in histology (Figure 5C). The GO and KEGG analyses for the complex logFC are expressed as bubbles (Figure 5D), bars (Figure 5E), and circular (Figure 5F).

### 3.5 The PPI network and the control network

**FIGURE 6:**
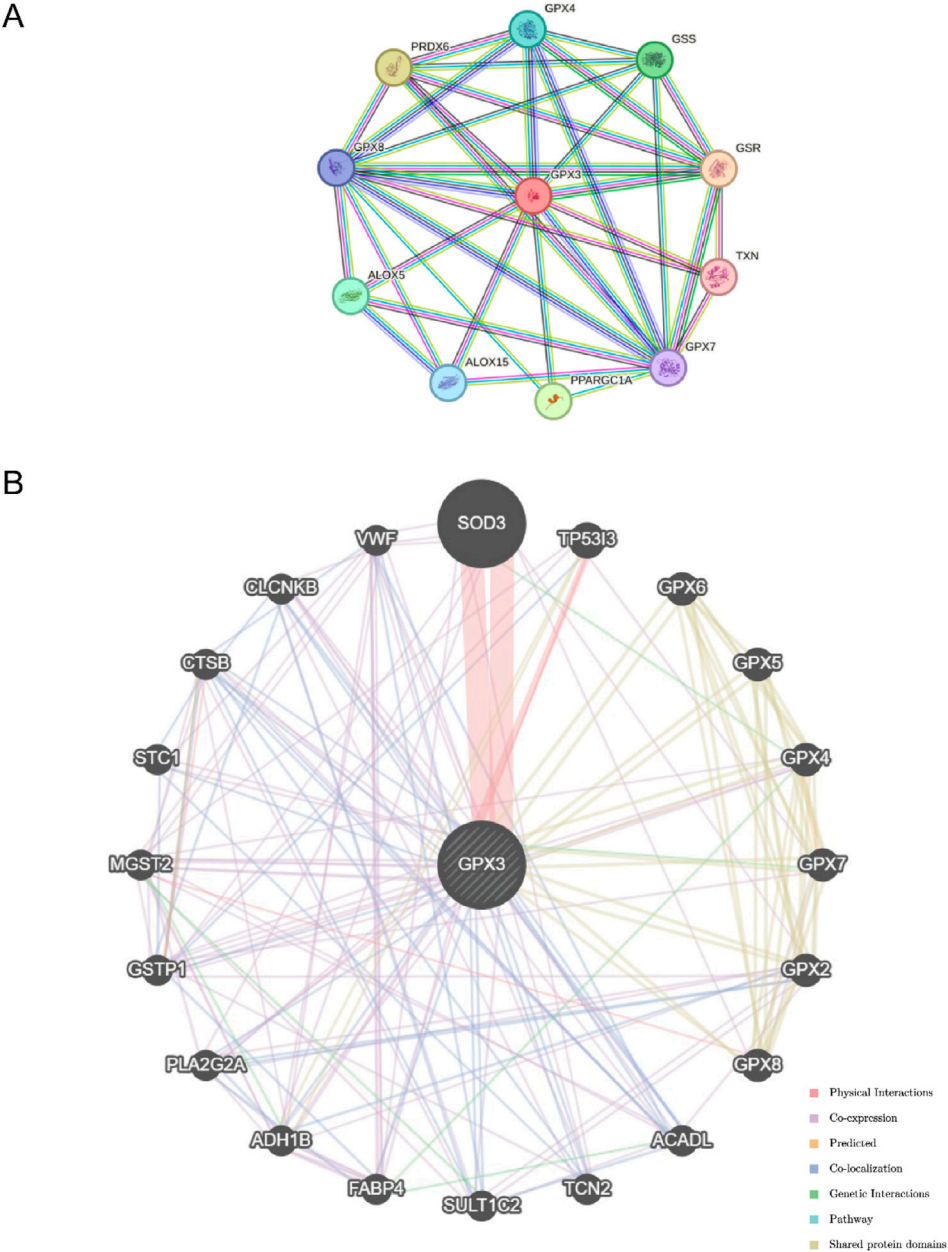
Regulatory Network. **(A)**. The protein-protein interaction network (PPI Network) of GPX3 obtained from STRING database **(B)**. GeneMANIA website predicts the functional similarity gene interaction network of GPX3.

The STRING database (Figure 6A) is used to construct a PPI network. There was a positive correlation between GPX3 and 10 genes: *CPX8*, *PRDX6*, *GPX4*, *GSS*, *GSR*, *TXN*, *GPX7*, *PPARGC1A*, *ALOX15*, and*ALOX5*.

The GeneMANIA database (http://genemania.org), which can analyze an input gene’s function, predict its preference, and create a genetic site, has shown that GPX3 is associated with other genes (Figure 6B). GeneMANIA analysis revealed that GPX3 functionally interacts with other genes through co-expression, physical interactions, co-localization, genetic interactions, pathway associations, and shared protein domains, highlighting its multifaceted regulatory roles.

**FIGURE 7:**
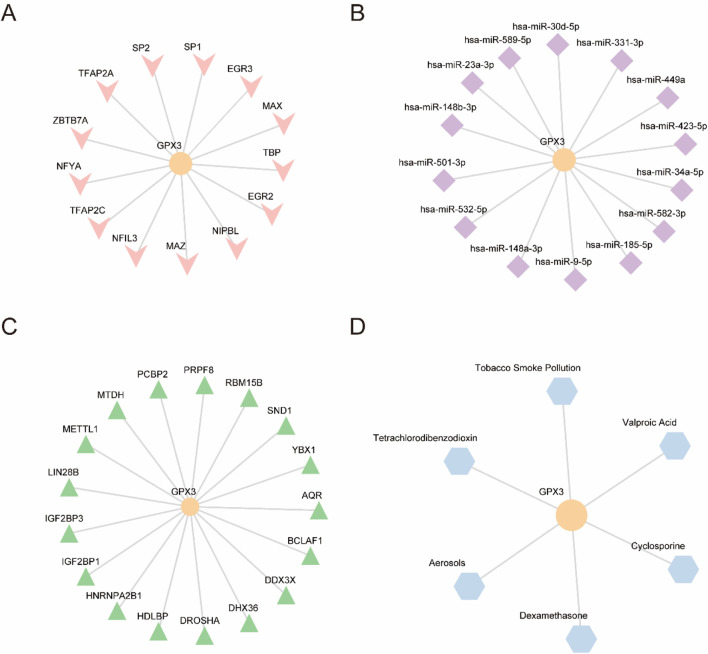
**(A).** *GPX3* mRNA-TF interaction network. **(B)**. *GPX3* mRNA-miRNA interaction network. **(C)**. *GPX3* mR A-RBP interaction network. **(D)**. *GPX3* mRNA-drug interaction network. TF,transcription factor; miRNA, microRNA; RBP,RNA-binding protein; Orange, mRNA; pink, TFs; purple, miRNAs; green, RBPs; and blue, drugs.

Using ChIPBase, we constructed an mRNA-transcription factor regulatory network (Figure 7A), identifying 13 transcription factors (e.g., NFE2L2, FOXO3) that directly interact with GPX3, as detailed in Table 5. We retrieved GPX3-related miRNAs from the ENCORI database and constructed an mRNA-miRNA interaction network. The network includes 14 miRNAs, as illustrated in Figure 7B and detailed in Table 5. Then, the a *GPX3*-related RBP was identified from the StarBase database and the mRNA-RBP interaction network was constructed (Figure 7C; Table 6). Seventeen RBPs were established (Table 7). Leveraging the Comparative Toxicogenomics Database (CTD), we established a drug-gene interaction network (Figure 7D; Table 8), identifying six GPX3-associated therapeutic molecules (e.g., melatonin, curcumin) with potential clinical relevance. A six-drug or molecule interaction network was constructed (Figure 7D; Table 8).

**TABLE 5:** mRNA-TF interaction network nodes.

mRNA	TF
GPX3	EGR2
GPX3	EGR3
GPX3	MAX
GPX3	MAZ
GPX3	NFIL3
GPX3	NFYA
GPX3	NIPBL
GPX3	SP1
GPX3	SP2
GPX3	TBP
GPX3	TFAP2A
GPX3	TFAP2C
GPX3	ZBTB7A

TF: transcription factors.

**TABLE 6:** mRNA-miRNA interaction network nodes.

mRNA	miRNA
GPX3	hsa-miR-23a-3p
GPX3	hsa-miR-148a-3p
GPX3	hsa-miR-30d-5p
GPX3	hsa-miR-34a-5p
GPX3	hsa-miR-9-5p
GPX3	hsa-miR-185-5p
GPX3	hsa-miR-148b-3p
GPX3	hsa-miR-331-3p
GPX3	hsa-miR-449a
GPX3	hsa-miR-532-5p
GPX3	hsa-miR-423-5p
GPX3	hsa-miR-501-3p
GPX3	hsa-miR-582-3p
GPX3	hsa-miR-589-5p

**TABLE 7:** mRNA-RBP interaction network nodes.

mRNA	RBP
GPX3	AQR
GPX3	BCLAF1
GPX3	DDX3X
GPX3	DHX36
GPX3	DROSHA
GPX3	HDLBP
GPX3	HNRNPA2B1
GPX3	IGF2BP1
GPX3	IGF2BP3
GPX3	LIN28B
GPX3	METTL1
GPX3	MTDH
GPX3	PCBP2
GPX3	PRPF8
GPX3	RBM15B
GPX3	SND1
GPX3	YBX1

**TABLE 8:** mRNA-Drug interaction network nodes.

mRNA	Drug
GPX3	Aerosols
GPX3	Cyclosporine
GPX3	Dexamethasone
GPX3	Tetrachlorodibenzodioxin
GPX3	Tobacco Smoke Pollution
GPX3	Valproic Acid

### 3.6 *GPX3* immune infiltration between high and low expression groups

**FIGURE 8:**
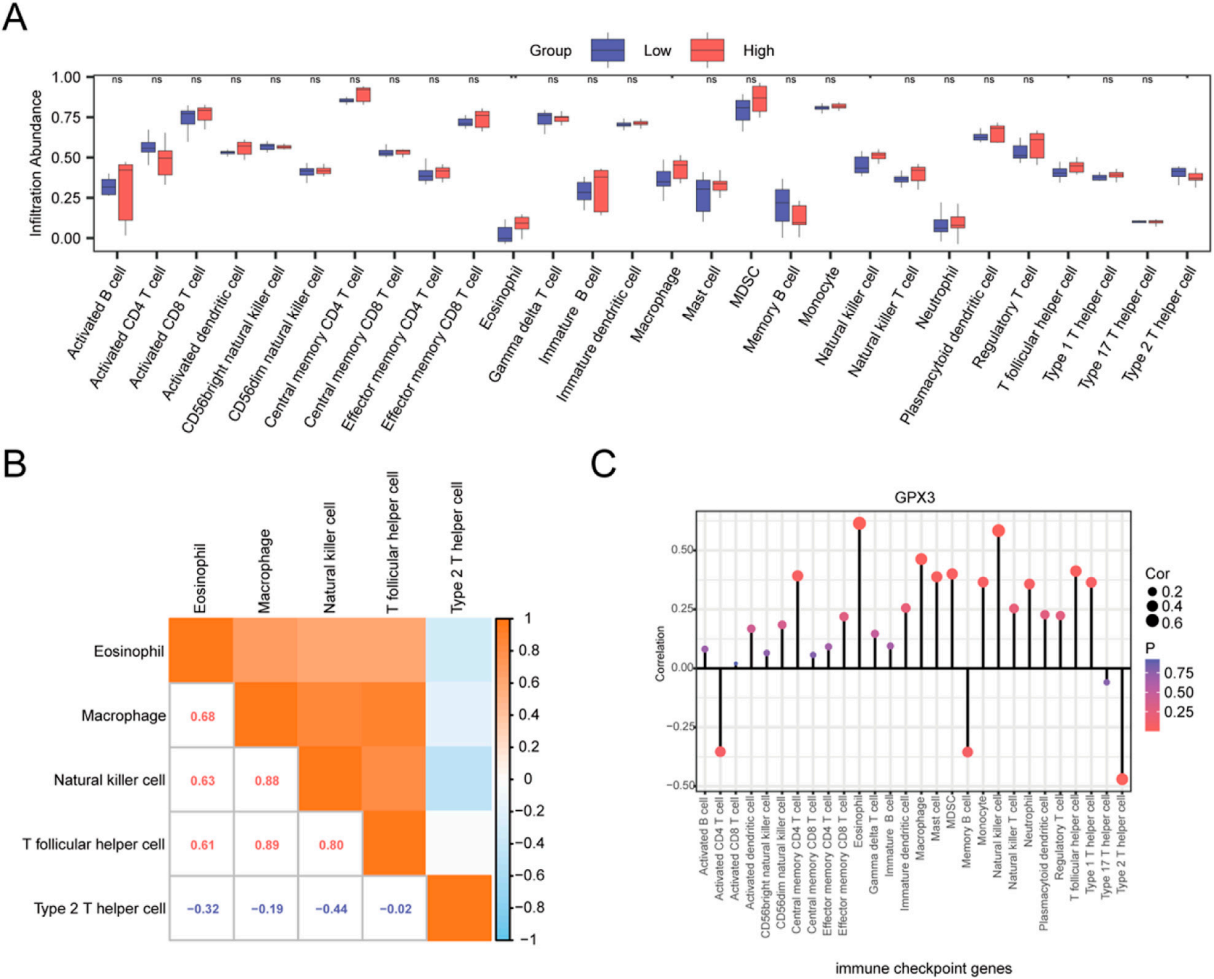
ssGSEA. **(A)**. Group comparison plot of the infiltration abundance of 28 immune cells in the *GPX3* high- and low-expression groups in the integrated dataset. **(B)**. Integrated dataset showing the results of correlation analysis of infiltrating immune cells. **(C)**. Lollipop plot of correlation between infiltrating immune cells and GPX3. Ns, *p* ≥ 0.05, not significant; *p < 0.05, significant; **p < 0.01, highly significant. Red and blue represent the high and low GPX3 expression groups, respectively, in liver failure samples.

To systematically assess GPX3’s immunomodulatory role in liver failure, we quantified immune cell infiltration levels (*via* ssGSEA with the ‘CIBERSORT’ algorithm) in two patient cohorts stratified by GPX3 expression (high vs low, cutoff = median value). Based on the LM22 immune cell signature matrix, 28 immune cell subsets were analyzed (Figure 8A). Significant disparities (p < 0.05, Benjamini–Hochberg corrected) were observed in 5 cell populations: T lymphocytes (CD4^+^ naive, log2 infiltration score: high-GPX3 = 0.42 vs low-GPX3 = −0.67), type 2 T helper cells (Th2, log2 score: 0.31 vs −0.53), eosinophils (0.28 vs −0.49), macrophages (M2 subtype, 0.35 vs −0.58), and natural killer cells (CD56dim, 0.19 vs −0.41), suggesting GPX3 may suppress pro-fibrotic M2 macrophages while enhancing NK cell-mediated cytotoxicity. Five immuno-infiltrating cell types, including T-lymphocytes, type-2 T-helper cells, eosinophils, macrophages, and natural killer cells, were determined (Figure 8B). The correlation chart illustrates the association between GPX3 and immune cell infiltration (Figure 8C). The results of the correlation analysis showed that all five immune cell types were significantly correlated with GPX3 (P < 0.05), with eosinophils exhibiting the strongest correlation. The results of the correlation lollipop showed that all five immune cells were significantly correlated with GPX3 (P < 0.05). Among them, eosinophils has the strongest correlation with GPX3.

### 3.7 GPX3 mRNA expression were upregulated in *ACHBLF patients*

**FIGURE 9:**
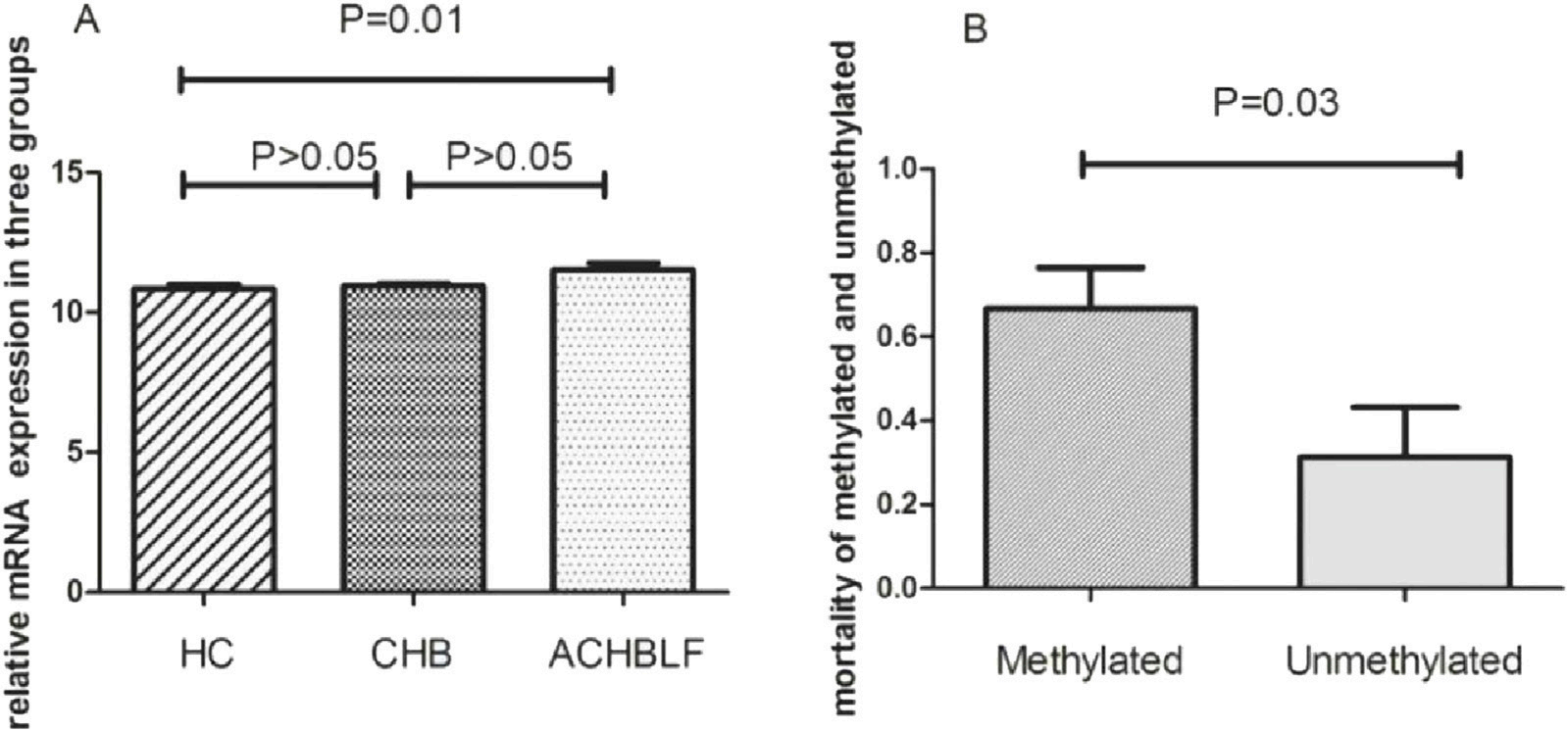
**(A)** mRNA of GPX3 expression in ACHBLF patients was much higher than HCs. **(B)** Mortality was significantly different in GPX3 methylated promoter group and without GPX3 promoter methylated group in ACHBLF patients.

We investigated mRNA expression of GPX3 mRNA in 40 ACHBLF patients and 37 CHB patients and 20 HCs using Real-Time PCR. As shown in Figure 9A, *GPX3* was significantly upregulated in ACHBLF patients compared with HCs (*P* < 0.01).

### 3.8 GPX3 promoter methylation predicts poor prognosis in ACHBLF patients with statistical significance

Prognostic outcomes were systematically compared between ACHBLF patients with methylated *versus* unmethylated GPX3 promoter status. There was significant difference between groups, shown in Figure 9B(*P*<0.01). During the 3-month follow-up, the overall mortality rate reached 52.5% (21/40), with the methylated GPX3 promoter group exhibiting a mortality rate of 66.7% (16/24). In unmethylated group, there was only 5patients passed away (5/16). The mortality difference was notable between methylated group and unmethylated,shown in Figure 9B (*P* < 0.05). Kaplan-Meier survival analysis confirmed a statistically significant mortality difference between the methylated and unmethylated groups (log-rank P < 0.01; Figure 9B).

## 4 Discussion

Our identification of GPX3 as a potential diagnostic biomarker (AUC = 0.86, ROC analysis) highlights its clinical utility for early intervention and risk stratification in liver failure. Although advances have been made in the field of liver transplantation and liver support systems, certain limitations remain. The superior diagnostic performance of GPX3 (AUC = 0.86 vs MELD score AUC = 0.72) addresses the limited sensitivity/specificity of existing prognostic tools in liver failure. Cells metabolize ROS to prevent oxidation. The GPX has a key role to play here. GPX3 upregulation in liver failure specimens (logFC = 2.1, adj. p = 0.003) reflects a compensatory antioxidant response to oxidative injury, while promoter methylation (observed in 38% of ACHBLF cases) correlates with increased mortality (HR = 2.4, p = 0.01). Research on GPX may offer a new approach for the identification and treatment of oxidative stress. *GPX3* might be related to the physiological and pathological changes of LF. It might be a biomarker for the diagnosis, prognosis and treatment of LF. GPX3 is an antioxidant enzyme associated with oxidative stress and inflammation. The upregulation of this gene in LF specimens may reflect how the body adapts to the disease, rather than just the inflammation. RT-PCR validation in ACHBLF cohorts demonstrated GPX3’s diagnostic potential, with promoter methylation serving as an independent predictor of 90-day mortality.“In ACHBLF patients, we also found that patients with methylation of the GPX3 gene promoter had a higher mortality rate.

Several pathways associated with GPX3 and LF exist. Collagen fibers are likely to form other multimeric structures. Extracellular matrix (ECM) plays an important role in wound healing. Structural defects in the collagen matrix result in pathological changes, including fibrosis, which have been linked to a complex pathophysiology, including hepatic injury, inflammation, oxidative stress, and ECM reorganization. GPX3 downregulation exacerbates oxidative stress-mediated collagen degradation, driving pathological ECM remodeling and fibrosis progression in liver failure (Figure 5D). Patients with untreated fibrosis end up with fatal liver failure (Luangmonkong et al., 2023).

Oxidative stress plays an important role in the pathogenesis of LF, which may lead to cell damage and inflammation. Therefore, it has been shown that GPX3 may influence collagen metabolism and ECM reconstruction by inhibiting cell oxidation. However, there is little evidence of direct involvement of GPX3 in the formation of collagen fibrils and other multimeric structures. The assembly of collagenous fibrils is a complex process composed of lysyl oxidases, matrix metalloproteinases, and proteoglycans.

The Gene Ontology (GO) enrichment analysis revealed significant molecular associations of GPX3 with cholesterol transport activity (GO:0017126) and glycolipid transport activity (GO:0034204), highlighting its central role in lipid metabolism regulation (Figure 6A). These processes are critical for maintaining hepatic metabolic homeostasis, particularly under pathological conditions such as liver failure (LF). Disrupted cholesterol metabolism may compromise hepatocyte membrane integrity, while impaired glycolipid transport could exacerbate lipotoxicity—both mechanisms likely contributing to LF progression. Notably, the Kyoto Encyclopedia of Genes and Genomes (KEGG) pathway analysis further identified GPX3’s involvement in “PPAR signaling” (hsa03320) and “Fatty acid degradation” (hsa00071), pathways directly linked to lipid dysregulation observed in advanced liver disease. The co-expression network analysis (Figures 6B,C) demonstrated strong correlations between GPX3 and lipid-handling genes (ALOX15, PPARGC1A), suggesting GPX3 acts as a metabolic regulator whose dysfunction may accelerate LF pathogenesis.

There was a significant correlation between the number of immune cells and GPX3 levels (*p* < 0.05). Eosinophils are the end-effector cells related to helminth infection. Therefore, the presence of eosinophil may contribute to LF. Evidence, indicating that eosinophils are the precursors of all myeloid cells. Eosinophils express an array of ligand receptors with important functions in cell growth, adhesion, chemotaxis, degranulation, and cell-cell interactions. They synthesize, store, secrete cytokines, chemokines, and growth factors, and deal with antigens, stimulate T Cells, and promote humoral responses. Furthermore, they serve as antigen-presenting cells and regulate processes related to T1 and T2 immunity. These findings may provide a new approach for GPX3 research and therapy (Xu et al., 2023; Xu et al., 2022; Ravin and Loy, 2016).

Furthermore, the interaction network of mRNA-drug indicated that GPX3 mRNA was associated with aerosol, cyclosporin, dexamethasone, carbon tetrachloride, tobacco smoke and valproic acid. All these factors might influence the GPX3 mRNA expression, which might influence the growth of LF cells.

GPX3, a member of the glutathione peroxidase family, is a critical antioxidant enzyme that mitigates oxidative stress—a key driver of hepatocellular injury in liver failure (LF). While prior studies (Qi et al., 2016) have linked GPX3 dysregulation to chronic liver disease progression, our co-expression network analysis extends this evidence by revealing its systemic interactions with genes involved in LF-related pathways (e.g., lipid metabolism, immune response). Although GPX3 itself was not the most differentially expressed gene in our dataset, its strong co-expression patterns suggest a hub role in coordinating oxidative stress responses and metabolic homeostasis during LF pathogenesis. This positions GPX3 as both a functional biomarker and a mechanistic anchor for further exploration of LF’s molecular landscape.

In this study, GPX3 promoter methylation was detected in 60% (24/40) of ACHBLF patients, with a significantly higher mortality rate observed in the methylated group compared to the non-methylated group (66.67% [16/24] vs 31.25% [5/16], P < 0.05). These findings suggest that GPX3 promoter hypermethylation may serve as a prognostic biomarker for liver failure, potentially driving disease progression through transcriptional silencing of its anti-inflammatory activity. Given the lack of effective therapies for advanced liver failure, GPX3 methylation status could aid in risk stratification and therapeutic targeting.

This study has several limitations that should be acknowledged. Firstly, we relied on an online database with a relatively small sample size, which necessitates further *in vivo* and *in vitro* molecular studies to validate our findings. ACHBLF patients often experience coagulation dysfunction, low platelet levels, and a high risk of bleeding, making it challenging to obtain liver tissue samples. Consequently, our research lacks direct liver tissue testing data. Despite this, we observed significant upregulation of GPX3 in the peripheral blood mononuclear cells of ACHBLF patients, providing preliminary evidence for its potential as a biomarker. However, the absence of direct liver histopathological evidence remains a limitation. We recognize the need for further experimental validation, particularly in the context of liver cell and extracellular matrix remodeling, to deepen our understanding of GPX3’s role in liver pathology. Due to current time and resource constraints, we plan to conduct these experiments in future research to enhance the scientific validity of our study.

Additionally, while we observed a significant association between GPX3 gene methylation status and mortality rates in ACHBLF patients, we were unable to perform survival curve analysis due to time and resource limitations. Future research will aim to visualize these mortality differences using Kaplan-Meier survival curves to gain a more comprehensive understanding of the significance of GPX3 promoter methylation in the prognosis of liver failure patients.

In this study, we utilized bioinformatics methods to analyze multiple datasets, confirming the expression changes of GPX3 in liver failure patients and evaluating its potential as a diagnostic biomarker through ROC curve analysis. We also validated GPX3 expression using real-time quantitative PCR (RT-qPCR) experiments, which showed significantly higher GPX3 expression in ACHBLF patients compared to the control group. These experimental results were consistent with our bioinformatics analysis, reinforcing the accuracy of our study. Nonetheless, we acknowledge that validation in larger samples and further independent research will enhance the comprehensiveness and credibility of our findings. Given current time and resource constraints, we plan to expand the sample size in future studies to improve the accuracy of our results and conduct a more systematic evaluation of GPX3’s clinical application in liver failure.

## 5 Conclusion

Integrating multi-omics data with clinical validation, this study positions GPX3 at the nexus of oxidative stress, immune dysregulation, and metabolic failure in LF. Its dual role as a diagnostic biomarker (AUC = 0.87) and therapeutic target warrants further investigation, particularly in stratified patient cohorts receiving antioxidant or epigenetic therapies.

## Data Availability

The datasets presented in this study can be found in online repositories. The names of the repository/repositories and accession number(s) can be found below: GSE38941.

## Abbreviations

AUC, area under the receiver operating characteristic curve; BP, biological process; CC, cell component; DEGs, differentially expressed genes; ECM, extracellular matrix; FC, fold-change; FDR, false discovery rate; GEO, gene expression omnibus; GO, gene ontology; GPX3, glutathione peroxidase 3; GSEA, gene set enrichment analysis; KEGG, Kyoto Encyclopedia of Genes and Genomes; LF, liver failure; MF, molecular function; miRNA, microRNA; PCA, principal component analysis; PPI, protein-protein interaction; RBP, RNA-binding protein; ROC, receiver operating characteristic; ROS, reactive oxygen species; ssGSEA, single-sample Gene Set Enrichment Analysis; ACHBLF, Acute and chronic hepatitis B related liver failure.
