# Supplementary material for: Liver failure diagnosis: key diagnostic biomarkers discovery and bioinformatic validation
Source: Front Genet. 2025 Apr 10;16:1554116. doi: 10.3389/fgene.2025.1554116 (PMC12020437; doi:10.3389/fgene.2025.1554116)
Supplement: Supplementary file 1 [file Supplementaryfile1.pdf]

gene
CPX8
PRDX6
GPX4
GSS
GSR
TXN
GPX7
PPARGC1A
ALOX15
ALOX5
